# Plasma levels of phosphorylated tau 181 are associated with cerebral metabolic dysfunction in cognitively impaired and amyloid-positive individuals

**DOI:** 10.1093/braincomms/fcab073

**Published:** 2021-04-15

**Authors:** Firoza Z Lussier, Andréa L Benedet, Joseph Therriault, Tharick A Pascoal, Cécile Tissot, Mira Chamoun, Sulantha Mathotaarachchi, Melissa Savard, Nicholas J Ashton, Thomas K Karikari, Juan Lantero Rodriguez, Anniina Snellman, Gleb Bezgin, Min Su Kang, Jaime Fernandez Arias, Yi-Ting Wang, Serge Gauthier, Henrik Zetterberg, Kaj Blennow, Pedro Rosa-Neto, for the Alzheimer’s Disease Neuroimaging Initiative

**Affiliations:** 1 Translational Neuroimaging Laboratory, The McGill University Research Centre for Studies in Aging, Montréal, QC, Canada; 2 Department of Psychiatry and Neurochemistry, Institute of Neuroscience & Physiology, The Sahlgrenska Academy, University of Gothenburg, Mölndal, Sweden; 3 Wallenberg Centre for Molecular and Translational Medicine, University of Gothenburg, Gothenburg, Sweden; 4 Maurice Wohl Clinical Neuroscience Institute, Institute of Psychiatry, Psychology & Neuroscience, King’s College London, London, UK; 5 NIHR Biomedical Research Centre for Mental Health & Biomedical Research Unit for Dementia at South London & Maudsley NHS Foundation, London, UK; 6 Turku PET Centre, University of Turku, Turku, Finland; 7 Alzheimer’s Disease Research Unit, The McGill University Research Centre for Studies in Aging, Montréal, QC, Canada; 8 Department of Neurodegenerative Disease, UCL Institute of Neurology, London, UK; 9 Clinical Neurochemistry Laboratory, Sahlgrenska University Hospital, Mölndal, Sweden; 10 UK Dementia Research Institute at UCL, London, UK; 11 Montréal Neurological Institute, Montréal, QC, Canada; 12 Department of Neurology and Neurosurgery, McGill University, Montréal, QC, Canada

**Keywords:** Alzheimer’s disease, plasma, phosphorylated tau, brain glucose metabolism, metabolic dysfunction

## Abstract

Alzheimer’s disease biomarkers are primarily evaluated through MRI, PET and CSF methods in order to diagnose and monitor disease. Recently, advances in the assessment of blood-based biomarkers have shown promise for simple, inexpensive, accessible and minimally invasive tools with diagnostic and prognostic value for Alzheimer’s disease. Most recently, plasma phosphorylated tau181 has shown excellent performance. The relationship between plasma phosphorylated tau181 and cerebral metabolic dysfunction assessed by [^18^F]fluorodeoxyglucose PET in Alzheimer’s disease is still unknown. This study was performed on 892 older individuals (297 cognitively unimpaired; 595 cognitively impaired) from the Alzheimer’s Disease Neuroimaging Initiative cohort. Plasma phosphorylated tau181 was assessed using single molecular array technology and metabolic dysfunction was indexed by [^18^F]fluorodeoxyglucose PET. Cross-sectional associations between plasma and CSF phosphorylated tau181 and [^18^F]fluorodeoxyglucose were assessed using voxelwise linear regression models, with individuals stratified by diagnostic group and by β-amyloid status. Associations between baseline plasma phosphorylated tau181 and longitudinal (24 months) rate of brain metabolic decline were also assessed in 389 individuals with available data using correlations and voxelwise regression models. Plasma phosphorylated tau181 was elevated in β-amyloid positive and cognitively impaired individuals as well as in apolipoprotein E ε4 carriers and was significantly associated with age, worse cognitive performance and CSF phosphorylated tau181. Cross-sectional analyses showed strong associations between plasma phosphorylated tau181 and [^18^F]fluorodeoxyglucose PET in cognitively impaired and β-amyloid positive individuals. Voxelwise longitudinal analyses showed that baseline plasma phosphorylated tau181 concentrations were significantly associated with annual rates of metabolic decline in cognitively impaired individuals, bilaterally in the medial and lateral temporal lobes. The associations between plasma phosphorylated tau181 and reduced brain metabolism, primarily in cognitively impaired and in β-amyloid positive individuals, supports the use of plasma phosphorylated tau181 as a simple, low-cost, minimally invasive and accessible tool to both assess current and predict future metabolic dysfunction associated with Alzheimer’s disease, comparatively to PET, MRI and CSF methods.

## Teaser

Lussier *et al.* report that plasma phosphorylated tau181 is associated cross-sectionally with CSF phosphorylated tau181 and [^18^F]fluorodeoxyglucose PET in 823 individuals from the Alzheimer’s Disease Neuroimaging Initiative cohort. The authors also report a significant association between baseline plasma p-tau181 and longitudinal change in brain glucose metabolism indexed by [^18^F]fluorodeoxyglucose PET.

## Graphical Abstract

**Graphical Abstract:**
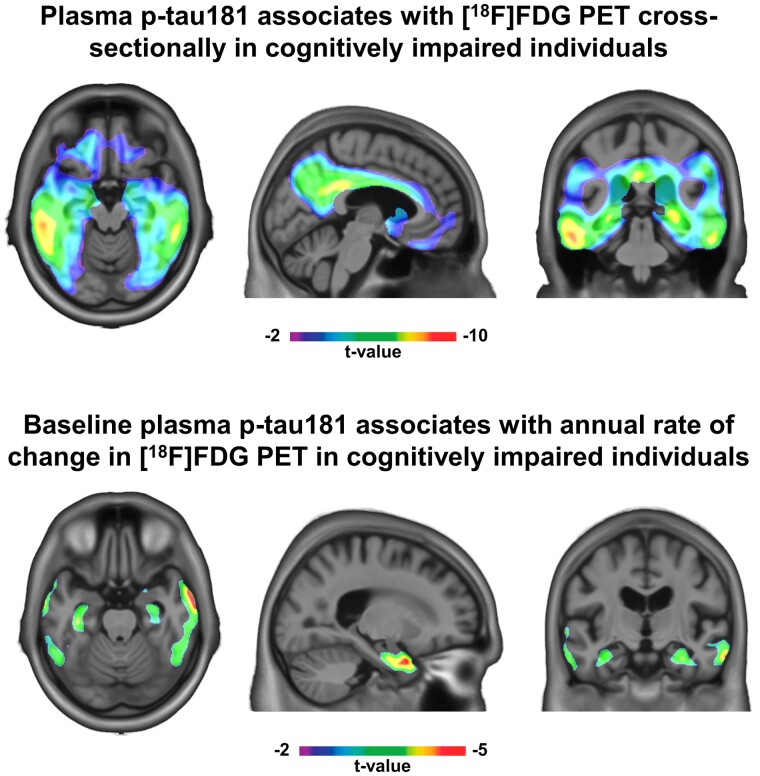

## Introduction

The pathognomonic signs of Alzheimer’s disease are the accumulation of β-amyloid (Aβ) and the aggregation of hyperphosphorylated tau into intraneuronal tangles.^1^ Alzheimer’s disease is also importantly characterized by brain glucose metabolism dysfunction and cerebral atrophy.^2^ As these pathological changes precede the appearance of clinical symptoms by many years,^3^ these pathologies may play an important role in both research and clinical trials for the screening, diagnosis and progression monitoring of Alzheimer’s disease.^4^

Currently, these biomarkers, i.e. Aβ, tau, glucose metabolism and brain atrophy, are primarily assessed through PET, MRI and CSF measures.^3^^,^^5^ However, the excessive cost, relative invasiveness and time-consuming nature of these methods obstruct their use in clinical practice.^6^ As such, given the need for more accessible Alzheimer’s disease biomarkers, blood-based biomarkers, such as measures of phosphorylated tau, Aβ42/40 ratio and neurofilament light protein,^7^ constitute a viable promise and warrant thorough investigation with regards to their specificity to Alzheimer’s disease.^8^

Phosphorylated tau is the principal component of neurofibrillary tangles and dystrophic neurites in Alzheimer’s disease. Tau protein phosphorylated at threonine-181 (p-tau181) has been examined in CSF,^9^ and it has been demonstrated that p-tau181 is highly specific for Alzheimer’s disease-related tau aggregation.^2^ Importantly, recent technological advancements have led to ultrasensitive assays of p-tau181 in blood samples (i.e. plasma and serum) using ultrasensitive immunoassays^10–14^ and mass spectrometry methods.^15^ Plasma p-tau181 levels have been shown to be strongly associated with brain tau pathology, significantly elevated in Alzheimer’s disease and differentiate the disease from other neurodegenerative disorders.^10–14^ However, to date the associations between plasma p-tau181 and Alzheimer’s disease-related brain metabolic dysfunction, a well-recognized pathophysiological process underlying Alzheimer’s disease, remains unknown.

In order to address this knowledge gap, the current study was designed to measure plasma p-tau181 levels and brain glucose metabolism as assessed by [^18^F]fluorodeoxyglucose (FDG) PET in participants of the Alzheimer’s Disease Neuroimaging Initiative (ADNI). The goal of the study is to examine (i) how the plasma biomarker compares to the CSF biomarker in terms of its association to [^18^F]FDG PET cross-sectionally and (ii) how baseline levels of plasma p-tau181 relate to longitudinal change in brain metabolic decline. We hypothesize that plasma p-tau181 performs similarly to CSF p-tau181 with regards to its relationship to brain metabolic dysfunction and that baseline plasma p-tau181 is able to predict reduction of brain metabolism over time.

## Materials and methods

### Study participants

The current study was based on data from the ADNI database. ADNI is a multicentre study launched in 2003 as a public-private partnership, led by Principal Investigator Michael W. Weiner, MD. ADNI’s primary goal is to test whether the combination of neuroimaging and biochemical biomarkers and clinical and neuropsychological assessments can be used for early detection and monitoring of Alzheimer’s disease dementia.^16^ The ADNI study was approved by local Institutional Review Boards of participating institutions, and informed written consent was provided by enrolled participants at each site. Full information regarding the ADNI inclusion/exclusion criteria is described elsewhere (http://adni.loni.usc.edu/, last accessed 15 February 2021). ADNI is a prospective cohort study that continues to recruit participants; this study was based on participants with available plasma p-tau181 data (data downloaded in June 2020).

The study population was classified into two diagnostic groups: cognitively unimpaired (CU) and cognitively impaired (CI) individuals. The CU classification was based on a CDR of 0; participants who had no cognitive dysfunction but reported subjective cognitive decline were analyzed together with CU, as per the National Institute of Aging-Alzheimer’s Association’s biological Alzheimer’s disease research framework.^2^ The CI group consisted of individuals that were clinically defined as having a mild cognitive impairment or Alzheimer’s disease dementia. Mild cognitive impairment and Alzheimer’s disease dementia classification followed the criteria described elsewhere.^16^^,^^17^ CSF p-tau181 and [^18^F]FDG PET data were matched with plasma p-tau181 data collected at the same ADNI study visit. Cross-sectional analyses were conducted on individuals who had available CSF and plasma measures of p-tau181 and PET measures of glucose metabolism ([^18^F]FDG) at the same ADNI study visit, which corresponded to *n *=* *823 participants [CU, *n *=* *262; CI, *n *=* *561 (mild cognitive impairment, *n *=* *426; Alzheimer’s disease, *n *=* *135)]. Longitudinal analyses were conducted on participants who had a baseline plasma p-tau181 assessment and a baseline and 24-month [^18^F]FDG PET assessment, which consisted of *n *=* *389 participants [CU, *n *=* *138; CI, *n *=* *251 (mild cognitive impairment, *n *=* *213; Alzheimer’s disease, *n *=* *38)]. A description of the cross-sectional and longitudinal sample selections can be found in Supplementary Fig. 1. The first available plasma p-tau181 measurement was used as the baseline time point for longitudinal analyses, as well as for age and diagnostic classification for cross-sectional and longitudinal analyses.

### Patient consent

The ADNI study was approved by the local Institutional Review Boards of all of the participating institutions. Informed written consent was provided by enrolled participants at each site.

### Plasma p-tau181 measurement

Blood samples were collected, shipped and stored as described by the ADNI Biomarker Core Laboratory (http://adni.loni.usc.edu/methods/, last accessed 15 February 2021). Plasma p-tau181 was analyzed with the Single Molecule Array (Simoa) technique, using a clinically validated in-house assay described previously.^10^ Plasma p-tau181 was measured on Simoa HD-X instruments (Quanterix, Billerica, MA, USA) in April 2020 at the Clinical Neurochemistry Laboratory, University of Gothenburg, Mölndal, Sweden. Plasma p-tau181 data were collected over 47 analytical runs. The assay precision was assessed by measuring two different quality control samples at the start and end of each run, resulting in within-run and between-run coefficients of variation of 3.3–11.6% and 6.4–12.7%, respectively. Out of 3762 ADNI samples, four were removed due to inadequate volumes. The remaining 3758 all measured above the assay’s lower limit of detection (0.25 pg/ml), with only six below the lower limit of quantification (1.0 pg/ml). Plasma p-tau181 measurements were downloaded from the ADNI database (accessed 2020-06-20).

### CSF p-tau181 measurement

CSF samples were collected by lumbar puncture, shipped and stored as described by the ADNI Biomarker Core Laboratory (http://adni.loni.usc.edu/methods/, last accessed 15 February 2021). CSF concentrations of p-tau181 were quantified using fully automated Elecsys immunoassays (Roche Diagnostics) at the ADNI Biomarker Laboratory at the University of Pennsylvania. The lower and upper technical limits for CSF p-tau181 were 8 and 120 pg/ml. Procedures have been described in detail previously.^18^^,^^19^

### MRI acquisition and processing

Pre-processed 3 T MRI T1-weighted magnetization-prepared rapid acquisition gradient-echo images were downloaded from the ADNI database; full information regarding ADNI acquisition and pre-processing protocols of MRI data can be found elsewhere (http://adni.loni.usc.edu/methods/mri-tool/mri-analysis/, last accessed 15 February 2021).^20^ Images underwent linear and non-linear registration to the ADNI template space, and all images were visually inspected to ensure proper alignment to the ADNI template.

### PET acquisition and processing

Pre-processed [^18^F]FDG and [^18^F]Florbetapir PET images were downloaded from the ADNI database; full information regarding ADNI acquisition and pre-processing protocols of PET data can be found elsewhere (http://adni.loni.usc.edu/methods/pet-analysis-method/pet-analysis/, last accessed 15 February 2021). Images underwent spatial normalization to the ADNI standardized space using the automatic registration of PET images to their corresponding T1-weighted image space as well as the linear and non-linear transformations from the T1-weighted image space to the ADNI template space. PET images were spatially smoothed to achieve a final resolution of 8 mm full width at half maximum and were visually inspected to ensure proper alignment to the ADNI template.

[^18^F]FDG and [^18^F]Florbetapir standardized uptake value ratio (SUVR) maps were generated using the pons and the full cerebellum as the reference region, respectively. For each participant, a global [^18^F]FDG SUVR value was estimated by averaging the SUVR from the angular gyrus, posterior cingulate and inferior temporal cortices.^21^ A global [^18^F]Florbetapir SUVR value was similarly estimated using the precuneus, prefrontal, orbitofrontal, parietal, temporal, anterior and posterior cingulate cortices.^21^ Amyloid-β (Aβ) positivity was determined for each participant by a global [^18^F]Florbetapir SUVR exceeding 1.11.^22^

### Statistical analyses

All non-imaging statistical analyses were performed using R v4.0.0. Voxelwise imaging statistical analyses were executed using the VoxelStats toolbox^23^ in MATLAB version 9.4. Subjects were considered outliers if their baseline plasma p-tau181 value was three standard deviations above the population mean, and their data were excluded. Comparing demographic and clinical characteristics between diagnostic groups was done using χ^2^ test with continuity correction for categorical variables, Mann–Whitney *U* test for non-normal continuous variables and one-way ANOVA for normal continuous variables. Correlations between plasma p-tau181 levels and demographic and clinical characteristics used Pearson’s correlation coefficient (*r*). All *P* values were two-tailed and *P* values <0.05 were considered significant.

Cross-sectional data were evaluated with correlations between log-transformed CSF and plasma p-tau181 concentrations using Pearson’s correlation coefficient, with subjects stratified by diagnostic group and Aβ status. Voxelwise linear regression models tested the cross-sectional associations between [^18^F]FDG PET uptake and both CSF and plasma p-tau181 concentrations, adjusting for age and sex, in diagnostic groups (with and without Aβ status stratification).

Longitudinal analyses investigated the associations between baseline plasma p-tau181 levels and longitudinal metabolic decline. Annual rates of change were calculated both for global [^18^F]FDG SUVR and voxelwise for [^18^F]FDG images by subtracting the baseline value from the 24-month follow-up value and normalizing by the time difference between time points, in years. Correlations and voxelwise linear regression models then tested the associations between the annual rate of change in metabolic decline (using [^18^F]FDG SUVR and images, respectively) and log-transformed baseline concentration of plasma p-tau181 and, adjusting for age and sex. Log-transformation of CSF and plasma p-tau181 measurements in pg/ml was used in all voxelwise analyses in order to reduce the skew of the distribution. Random field theory with a cluster threshold of *P *<* *0.001 was used to correct voxelwise analyses for multiple comparisons.^24^

### Data availability

The dataset supporting the conclusions of this article is available in full on the ADNI site, at http://adni.loni.usc.edu/data-samples/access-data/ (last accessed 15 February 2021).

## Results

### Demographic characteristics

A total of 823 participants was included in the cross-sectional dataset, while 389 participants were included in the longitudinal dataset. In total, we studied unique 892 participants across both datasets, as we included individuals in the longitudinal dataset who did not have an available CSF p-tau181 assessment. From the unique participants, 297 were classified into the CU group and 595 into the CI group. Demographic and clinical characteristics are summarized for both datasets stratified by diagnostic group in Table 1.

**Table 1:** Demographic and clinical characteristics of the samples

Characteristic	Cross-sectional dataset (*n *=* *823)		Longitudinal dataset (*n *=* *389)	
Diagnostic group	CU	CI	CU	CI
*n*	262	561	138	251
Age (median [IQR])^a^	73.00 [68.52–78.56]	72.81 [67.09–77.60]	75.04 [69.93–80.38]	71.47 [65.98–77.30]
Males (*n*, %)^b^	120 (45.8)	317 (56.5)	75 (54.3)	138 (55.0)
Education (median [IQR])^a^^,^^b^	16.00 [15.00–18.00]	16.00 [14.00–18.00]	16.00 [16.00–19.00]	16.00 [14.00–18.00]
*APOE* ε4 carriers (*n*, %)^a^^,^^b^	75 (28.6)	296 (52.8)	35 (25.4)	127 (50.6)
MMSE (median [IQR])^a^^,^^b^	29.00 [29.00–30.00]	28.00 [25.00–29.00]	29.00 [28.25–30.00]	28.00 [26.00–29.00]
Clinical Dementia Rating sum of boxes score (CDRSB) (median [IQR])^a^^,^^b^	0.00 [0.00–0.00]	1.50 [1.00–3.00]	0.00 [0.00–0.00]	1.50 [1.00–2.50]
Plasma p-tau181 (median [IQR])^a^^,^^b^^,^^c^	13.54 [9.32–18.15]	18.08 [11.91–24.39]	13.93 [9.72–19.08]	15.51 [10.94–24.80]
CSF p-tau181 (median [IQR])^b^^,^^c^	19.67 [15.49–26.61]	25.27 [18.04–36.12]	–	–
Aβ+ (*n*, %)^a^^,^^b^	52 (19.8)	282 (50.3)	28 (20.3)	114 (45.4)
	(2 N/A)	(2 N/A)	(2 N/A)	(4 N/A)
[^18^F]Florbetapir SUVR (median [IQR])^a^^,^^b^	0.96 [0.89–1.07]	1.11 [0.94–1.28]	0.96 [0.90–1.06]	1.07 [0.94–1.24]
	(2 N/A)	(2 N/A)	(2 N/A)	(4 N/A)
[^18^F]FDG SUVR (mean (SD))^a^^.b^	1.80 (0.20)	1.67 (0.26)	1.78 (0.19)	1.72 (0.24)

The demographic and clinical characteristics for participants in the cross-sectional and longitudinal datasets are presented, stratified by cognitive status. Normal variables were summarized using mean and standard deviation, while non-normal variables were summarized using median and interquartile range. Statistical differences between the cognitively unimpaired and impaired groups were tested for both datasets, using χ^2^ test with continuity correction for categorical variables, Mann–Whitney *U* test for non-normal continuous variables, and one-way ANOVA for normal continuous variables. Missing data regarding amyloid PET is reported for each group in both datasets in the table as N/A.

a Statistically significant difference between groups in the longitudinal dataset (*P *<* *0.05).

b Statistically significant difference between groups in the cross-sectional dataset (*P *<* *0.05).

c Measured in pg/ml.

Aβ = amyloid-β; CDR = clinical dementia rating; CI = cognitively impaired; CU = cognitively unimpaired; IQR = interquartile range; MMSE = mini-mental state examination; SUVR = standardized uptake value ration; SD = standard deviation.

Of the 892 unique individuals, 476 (53.4%) were male and median age at baseline plasma collection was 73.0 years (interquartile range 67.9–78.1). CI individuals were significantly younger (*P *<* *0.001) than CU participants only in the longitudinal dataset, whereas significantly more CI individuals were male (*P *=* *0.005) only in the cross-sectional dataset. CU individuals had more years of education (*p*_cross_ = 0.016, *p*_long_ = 0.027) than CI individuals in both datasets. As expected, in both datasets, CI individuals had significantly worse performance in cognitive tests (mini-mental state examination and CDR; *p*_cross_ < 0.001, *p*_long_ < 0.001 for both tests), higher amyloid load indexed by [^18^F]Florbetapir SUVR (*p*_cross_ < 0.001, *p*_long_ < 0.001), lower brain metabolism indexed by [^18^F]FDG SUVR (*p*_cross_ < 0.001, *p*_long_ = 0.011), and significantly more CI individuals were *APOE* ε4 carriers (*p*_cross_ < 0.001, *p*_long_ < 0.001) and Aβ-positive (*p*_cross_ < 0.001, *p*_long_ < 0.001).

**Figure 1:**
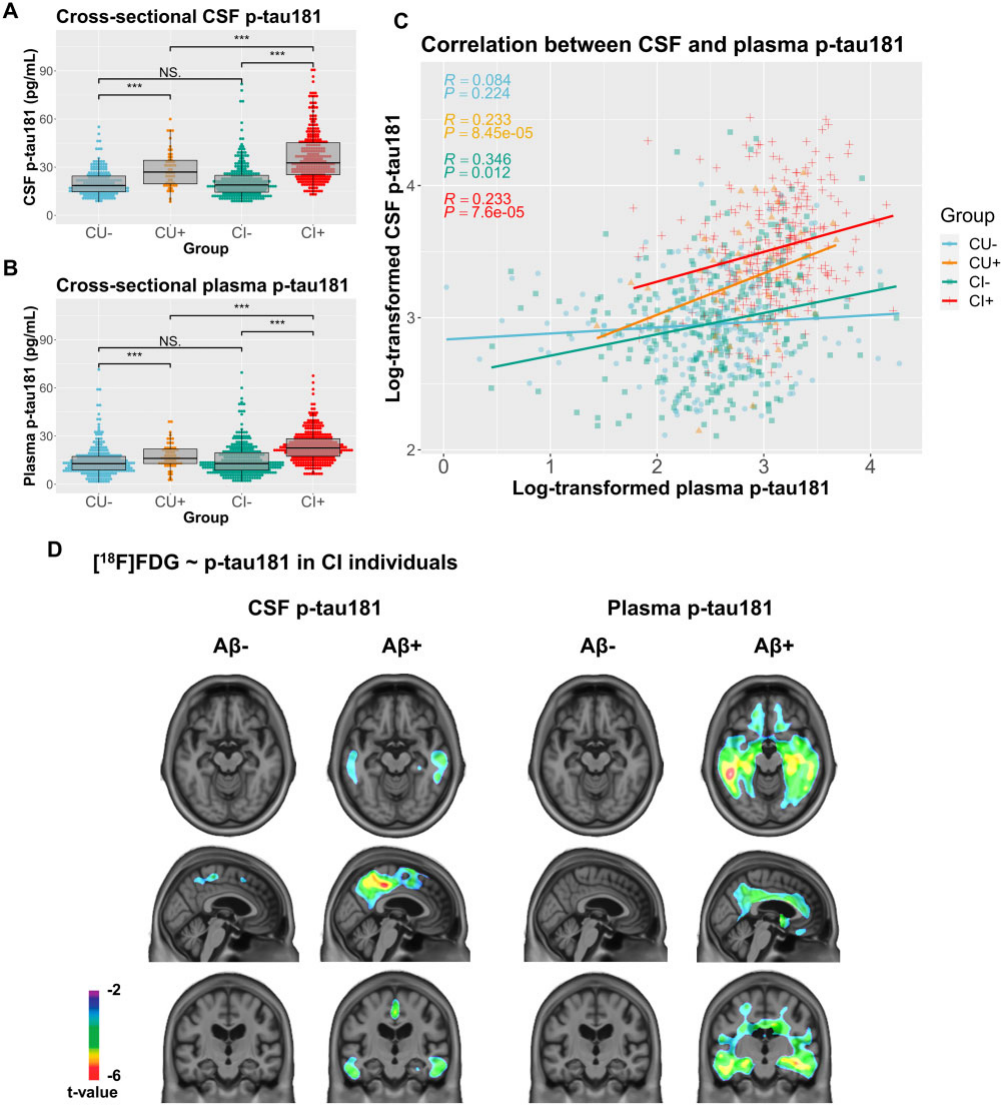
**Cross-sectional associations between plasma and CSF p-tau181 and between p-tau181 and [^18^F]FDG SUVR.** (**A**) CSF p-tau181 levels in pg/ml were compared in individuals in the cross-sectional dataset, stratified by both their cognitive status cognitively unimpaired (CU) or impaired (CI)] and Aβ status (+ or −), using Mann–Whitney *U* test. Significant differences in CSF p-tau181 were found between the CU- and CU+ groups (*P* < 0.001), the CU+ and CI+ groups (*P* < 0.001), and the CI− and CI+ groups (*P* < 0.001). (**B**) Similarly, plasma p-tau181 levels in pg/ml were compared cross-sectionally in individuals stratified by both cognitive and Aβ status. Significant differences in plasma p-tau181 were found between the CU− and CU+ groups (*P* < 0.001), the CU+ and CI+ groups (*P* < 0.001), and the CI− and CI+ groups (*P* < 0.001). (**C**) Pearson’s correlation coefficient (*r*) was computed for associations between log-transformed CSF levels and log-transformed plasma p-tau181 levels in individuals in the cross-sectional dataset stratified by cognitive and Aβ status. These measures were significantly positively correlated in the CU+ (*r* = 0.233, *P *=* *8.45e−05), CI− (*r* = 0.346, *P *=* *0.012), and CI+ groups (*r* = 0.233, *P *=* *7.6e−05), but not in the CU− group (*r* = 0.084, *P *=* *0.224). (**D**) Voxelwise linear regressions were performed to assess associations between log-transformed CSF p-tau181 and plasma p-tau181 in CI individuals stratified by Aβ status, adjusting for age and sex. Negative associations between CSF p-tau181 and [^18^F]FDG SUVR were observed bilaterally in CI Aβ+ individuals in the inferior temporal, posterior cingulate and precuneus (peak t-value of −5.78). Negative associations were found in similar brain regions between [^18^F]FDG uptake and plasma p-tau181 levels among CI Aβ+ participants (peak t-value of −6.03). Voxelwise results were corrected for multiple comparisons.

**Figure 2:**
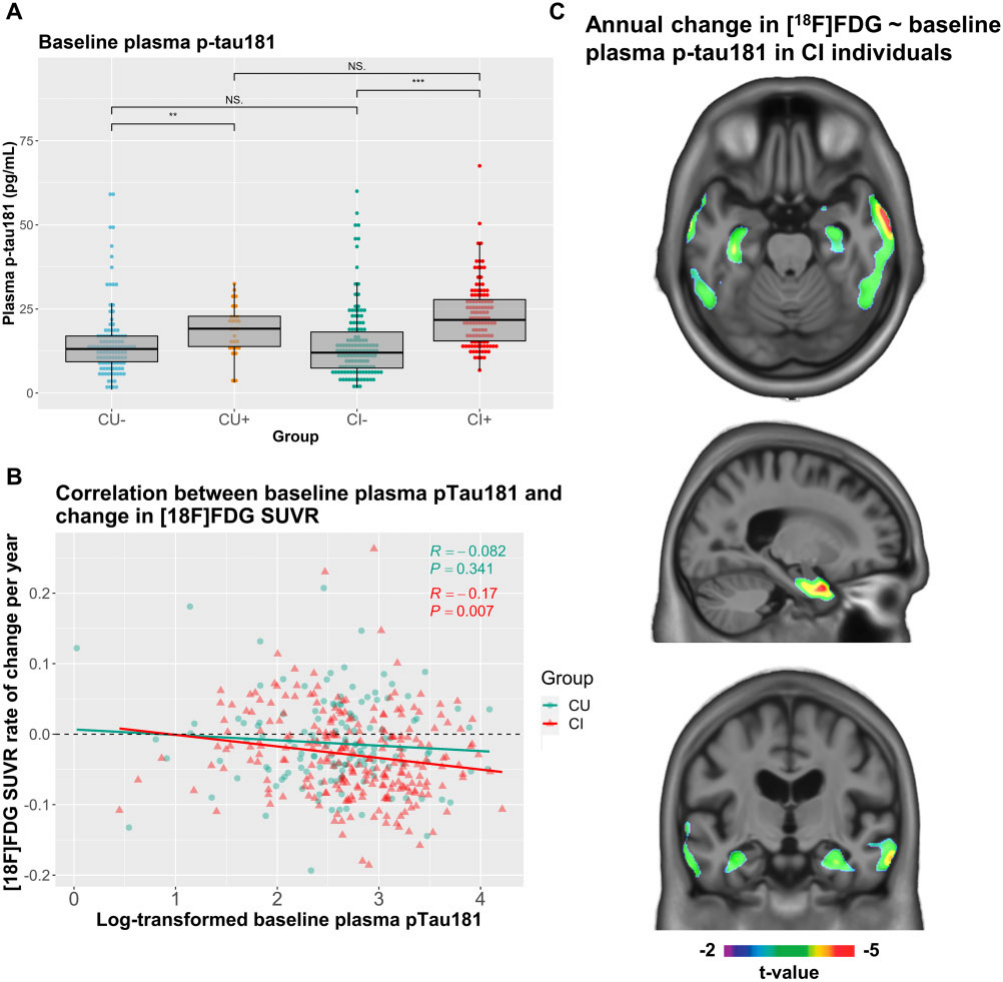
**Associations between baseline levels of plasma p-tau181 and longitudinal change in [^18^F]FDG SUVR.** (**A**) Baseline plasma p-tau181 levels in pg/ml were compared in individuals in the longitudinal dataset, stratified by both their cognitive status (cognitively unimpaired (CU) or impaired (CI)) and Aβ status (+ or −), using Mann–Whitney *U* test. Significant differences in baseline plasma p-tau181 levels were found between the CU− and CU+ groups (*P* < 0.001), and the CI− and CI+ groups (*P* < 0.001). (**B**) Pearson’s correlation coefficient (*r*) was computed for associations between log-transformed baseline plasma p-tau181 levels and annual rate of change in global [^18^F]FDG SUVR in individuals in the longitudinal dataset stratified by cognitive status. Log-transformed baseline plasma p-tau181 and change in global [^18^F]FDG SUVR were not significantly correlated in CU individuals (*r* = 0.082, *P *=* *0.341), but were significantly negatively correlated in CI individuals (*r* = −0.17, *P *=* *0.007). (**C**) Voxelwise linear regression models were used to investigate associations between baseline log-transformed plasma p-tau181 and annual rate of change in [^18^F]FDG SUVR in CI individuals in the longitudinal dataset, adjusting for age and sex. Significant associations were found in the medial and lateral temporal lobes (peak t-values of −5.01). Voxelwise results were corrected for multiple comparisons.

CI individuals had significantly elevated (*P *<* *0.001) levels of CSF p-tau181 compared with CU individuals in the cross-sectional dataset. Plasma p-tau181 concentrations were significantly higher (*P *<* *0.001) in CI individuals in both cross-sectional and longitudinal datasets. Considering the 892 unique participants at baseline, plasma p-tau181 levels were significantly higher in males (*P *=* *0.007), irrespective of diagnosis. Plasma p-tau181 was also highly significantly elevated in *APOE* ε4 carriers (*P *<* *0.0001) and in Aβ+ individuals (*P *<* *0.0001). Furthermore, plasma p-tau181 was positively associated with age (*r* = 0.17, *P *=* *3.7e−07), CDR sum of boxes score (*r* = 0.28, *P *<* *2.2e−16) and [^18^F]Florbetapir SUVR (*r* = 0.36, *P *<* *2.2e−16), and negatively associated with education (*r* = −0.09, *P *=* *0.006) and mini-mental state examination score (*r* = −0.26, *P *=* *6.6e−15). When stratifying CU and CI individuals of the cross-sectional dataset by Aβ status, we observed significant differences in CSF and plasma p-tau181 levels between CU Aβ− and CU Aβ+ groups, CI Aβ− and CI Aβ+ groups and CU Aβ+ and CI Aβ+ groups (*P *<* *0.001), but not CU Aβ− and CI Aβ− groups (Fig. 1A and B). Similarly, in the longitudinal dataset, we observed significant differences in baseline plasma p-tau181 levels between CU Aβ− and CU Aβ+ groups (*P *<* *0.01) and CI Aβ− and CI Aβ+ groups (*P *<* *0.001; Fig. 2A).

### Plasma p-tau181 associates cross-sectionally with CSF p-tau181 and brain metabolism

In the cross-sectional dataset, increased concentrations of plasma p-tau181 were correlated with greater CSF p-tau181 levels in CU Aβ+ (*r* = 0.233, *P *=* *8.45e−05), CI Aβ− (*r* = 0.346, *P *=* *0.012) and CI Aβ+ (*r* = 0.233, *P *=* *7.6e−05), but not in CU Aβ− individuals (*r* = 0.084, *P *=* *0.224; Fig. 1C). At the voxel level, linear regression models found no significant associations in CU individuals between [^18^F]FDG uptake and CSF p-tau181 whereas for plasma p-tau181, negative associations were observed in very small clusters in the anterior cingulate and left temporal and parietal lobes (results not pictured, peak t-value of −4.82). Within CI individuals, stratified by Aβ status, significant negative associations between CSF p-tau181 and [^18^F]FDG retention were observed in CI Aβ+ individuals bilaterally in the inferior temporal, posterior cingulate and precuneus (Fig. 1D; peak t-value of −5.78). Significant negative associations were also found between [^18^F]FDG uptake and plasma p-tau181 levels among CI Aβ+ participants in more widespread temporal lobe clusters, and in the anterior and posterior cingulate, precuneus and orbitofrontal cortices (peak t-value of −6.03). Associations between both CSF and plasma p-tau181 and [^18^F]FDG uptake in all CI participants without stratification are shown in Supplementary Fig. 2.

### Baseline plasma p-tau181 is associated with longitudinal decrease in brain metabolism

In the longitudinal dataset, the mean [^18^F]FDG PET follow-up time was 23.8 (±1.67) months. We calculated the annual rate of change in brain metabolism for global [^18^F]FDG SUVR, as well as the annual rate of change in every brain voxel. Average rate of change in global [^18^F]FDG SUVR was −0.024 (±0.062; negative symbol representing a reduction in [^18^F]FDG uptake) per year, and the rate of brain metabolic decline was significantly higher (*P *=* *0.011) in CI individuals (−0.029 ± 0.061) than in CU individuals (−0.013 ± 0.062). Average [^18^F]FDG voxelwise annual rate of change is shown in Supplementary Fig. 3 and is highest (i.e. more negative values representing more pronounced metabolic decline) in the posterior cingulate, precuneus, temporal, and medial and lateral prefrontal cortices.

Correlations between baseline concentration of plasma p-tau181 and annual rate of change in [^18^F]FDG SUVR was statistically significant and negative within CI individuals (*r* = −0.17, *P *=* *0.007), but not significant in CU individuals (Fig. 2B). When stratifying individuals by both diagnostic group and Aβ status, correlations were statistically significant only in the CU Aβ+ group (*r* = −0.4, *P *=* *0.035) (Supplementary Fig. 4). Voxelwise associations between baseline plasma p-tau181 and rate of change in [^18^F]FDG SUVR did not survive correction for multiple comparisons in CU individuals. In CI individuals, baseline plasma p-tau181 predicted rate of change of [^18^F]FDG SUVR in the bilateral medial and lateral temporal lobes (Fig. 2C, peak t-value of −5.01).

## Discussion

In this study, we provide not yet reported evidence for associations between plasma measures of p-tau181 and cerebral hypometabolism as assessed by [^18^F]FDG PET. Our main findings were that cross-sectionally, plasma p-tau181 is associated with the metabolic signatures of Alzheimer’s disease. Moreover, in cognitively impaired individuals, levels of plasma p-tau181 at baseline are associated with longitudinal metabolic decline. Taken together, our study suggests that plasma p-tau181 may provide a cost-effective and minimally invasive method to assess existing disease pathophysiology highly associated with metabolic dysfunction.

We found that plasma p-tau181 was higher in males and *APOE* ε4 carriers, which to our knowledge is a finding that has not yet been described. We also observed plasma p-tau181 to be significantly associated with older age, fewer years of education, an elevated global cortical composite measure of Aβ-PET, and worse performance on cognitive scores, which, with the exception of education, concur with earlier studies on plasma p-tau181.^10–13^^,^^25^ As previously described, in our cross-sectional analyses, plasma p-tau181 was correlated with CSF p-tau181.^10–12^ Moreover, in agreement with previous research, plasma levels of p-tau181 in our sample were significantly elevated in cognitively impaired individuals, as well as in Aβ+ individuals independent of their cognitive status.^10–13^

Our cross-sectional analyses indicated that plasma p-tau181 levels and metabolic dysfunction were associated in the temporal, anterior and posterior cingulate, precuneus and orbitofrontal cortices in CI Aβ+ individuals. Interestingly, one can speculate that the small clusters in the anterior corpus callosum present in CU individuals may indicate a link between white matter energetic abnormalities in early states of the disease.^26^ Importantly, in both CU and CI groups, higher plasma p-tau181 levels were linked to Aβ status. Furthermore, baseline plasma and CSF p-tau181 had highly similar associations with [^18^F]FDG PET, with higher correlations in individuals on the Alzheimer continuum (i.e. cognitively impaired amyloid-positive individuals) and in similar brain regions. This indicates that glucose metabolism associates with abnormal tau phosphorylation at threonine-181 measured in either blood or CSF.

Longitudinally, we found that baseline concentrations of plasma p-tau181 were significantly associated with annual rate of metabolic decline assessed by a decrease in global [^18^F]FDG SUVR, within CI and CU Aβ+ individuals, although the correlation coefficient was low for the CI group (*r* = −0.17, *P *=* *0.007). Voxelwise analysis, which provides additional regional information, conducted in the CI group revealed that plasma p-tau181 was significantly associated with annual rate of change in [^18^F]FDG uptake in the medial and lateral lobes after multiple comparison correction. Together, these results support the concept that elevated plasma p-tau181 implies the presence of worsening neurodegeneration.

In our results, the topography of hypometabolism was consistent with brain regions that are known to be affected by Alzheimer’s disease. Specifically, metabolic dysfunction in the posterior cingulate gyrus, precuneus, and medial and lateral temporal lobes are commonly observed in amnestic mild cognitive impairment and Alzheimer’s disease dementia.^27–29^ Moreover, the posterior cingulate gyrus, precuneus and medial and lateral temporal lobes are brain regions that are affected by significant tau aggregation in Alzheimer’s disease.^30^^,^^31^ Metabolic dysfunction in these regions is further associated with cognitive decline as well as increased risk of progression to dementia.^32^ Because tau aggregation as measured by PET^33^ and by CSF^34^ is tightly associated with brain metabolism, the results of our study suggest that plasma p-tau181 can serve as a less invasive and more accessible measure of Alzheimer’s disease-related cerebral metabolic dysfunction.

Neurodegeneration biomarkers in isolation are neither sensitive nor specific to Alzheimer’s disease.^2^ In both cross-sectional and longitudinal analyses, we found little associations between plasma p-tau181 and [^18^F]FDG PET in CU individuals. This finding is consistent with the observation that metabolic dysfunction is tightly related to cognitive decline^35^ and thus significant metabolic decline is more commonly observed in individuals with cognitive impairment. Furthermore, we conducted stratified analyses of relationships between plasma p-tau181 and [^18^F]FDG PET in Aβ+ and Aβ− individuals. In individuals on the Alzheimer’s disease continuum (Aβ+), we observed significantly more pronounced cross-sectional associations between plasma p-tau181 and [^18^F]FDG PET in regions vulnerable to hypometabolism in Alzheimer’s disease. However, in individuals who were not on the Alzheimer’s disease continuum (Aβ−), we did not observe associations between plasma measures of p-tau181 and brain metabolic dysfunction. This was observed for both CU and CI Aβ− individuals. These results are consistent with accepted disease models in which (detectable) Aβ aggregation occurs upstream of (detectable) tau aggregation.^3^^,^^36^ Taken together, these results suggest that the specificity of plasma p-tau181 for AD-type pathology^10^^,^^11^^,^^37^ provides important information about the aetiology of neurodegeneration and corresponding cognitive decline.

Associations between plasma measures of p-tau181 and [^18^F]FDG PET have important clinical implications. [^18^F]FDG PET is a commonly employed test in the differential diagnosis of individuals with cognitive impairment.^38^ Brain metabolism, as indexed with [^18^F]FDG PET, correlates with cognitive function^35^ and reduced brain metabolism constitutes an important risk for clinical progression to dementia.^39^ [^18^F]FDG PET abnormalities are also observed before MRI atrophy,^40^ suggesting that [^18^F]FDG PET is a sensitive marker of neurodegeneration. Previous studies have investigated the relationship between available plasma markers of neurodegeneration (such as plasma neurofilament light) and FDG PET, both cross-sectionally and longitudinally.^7^ As tau hyperphosphorylation is believed to precede the changes in cerebral metabolism according to the β-amyloid pathology, tau pathology, and neurodegeneration (ATN) framework,^3^ we aimed to investigate this relationship with a plasma biomarker which shows earlier abnormality during disease progression. Therefore, given our results in this analysis, plasma measures of p-tau181 show potential as a simple tool for the diagnosis and monitoring of AD, as well as for the screening of individuals for disease-modifying clinical trials.

The validity of our results is potentially influenced by methodological limitations. First, we only included ADNI participants with a 24-month follow-up [^18^F]FDG PET relative to plasma p-tau181 assessment as imaging data was not consistent at later time points (i.e. at 36 and 48 months). As a consequence, we may have observed stronger and more compelling associations between plasma p-tau181 and [^18^F]FDG PET, as accepted biomarker models of Alzheimer’s disease demonstrate that tau accumulation occurs upstream of metabolic dysfunction and neurodegeneration.^3^ Moreover, the ADNI cohort, from which all subjects in this study were selected, does not encompass individuals with neurodegenerative or tau-related diseases other than Alzheimer’s disease. Therefore, it is not known how plasma p-tau181 may perform in predicting current and future metabolic dysfunction in other neurodegenerative diseases. Further studies should conduct similar analyses in other more varied observational cohorts, as well as track brain metabolism through [^18^F]FDG PET over a longer time frame.

Our study provides evidence for associations between plasma measures of p-tau181 and brain metabolic dysfunction as measured by [^18^F]FDG PET. Subgroup analyses revealed more widespread associations in CI individuals as compared to CU individuals. Moreover, extensive associations were observed in Aβ+ individuals, whereas no associations between plasma p-tau181 and [^18^F]FDG PET were observed in Aβ− individuals. Finally, baseline levels of plasma p-tau181 were associated with rates of metabolic decline in CI individuals. Together, our results suggest that plasma p-tau181 provides interrelated information to [^18^F]FDG PET in the differential diagnosis of individuals with cognitive impairment and may be useful to predict metabolic dysfunction associated with Alzheimer’s disease.

## Supplementary material

Supplementary material is available at *Brain Communications* online.

## Supplementary Material

fcab073_Supplementary_Data

## Abbreviations

Aβ: β-amyloid
ADNI: Alzheimer’s Disease Neuroimaging Initiative
*APOE*: apolipoprotein E
CDR: clinical dementia rating
CI: cognitively impaired
CU: cognitively unimpaired
FDG: fluorodeoxyglucose
p-tau181: phosphorylated tau at threonine 181
SUVR: standardized uptake value ratio
