# Supplementary material for: Plasma levels of phosphorylated tau 181 are associated with cerebral metabolic dysfunction in cognitively impaired and amyloid-positive individuals
Source: Brain Commun. 2021 Apr 15;3(2):fcab073. doi: 10.1093/braincomms/fcab073 (PMC8088291; doi:10.1093/braincomms/fcab073)
Supplement: fcab073_Supplementary_Data [file fcab073_supplementary_data.pdf]

Supplementary Material to: Plasma levels of phosphorylated tau 181 are associated with cerebral metabolic dysfunction in cognitively impaired and amyloid-positive individuals, by Lussier *et al.*

Contents:

Supplementary Figure 1: Description of the selection of current study's cross-sectional and longitudinal datasets

Supplementary Figure 2: Voxelwise associations between CSF and plasma p-tau181 and [^{18}F]FDG SUVR in all cognitively impaired individuals

Supplementary Figure 3: Average voxelwise annual rate of change in [^{18}F]FDG SUVR for participants in the longitudinal dataset

Supplementary Figure 4: Correlation between plasma p-tau181 and change in [^{18}F]FDG SUVR according to cognitive and Aβ status

Supplementary Figure 1: Description of the selection of current study's cross-sectional and longitudinal datasets

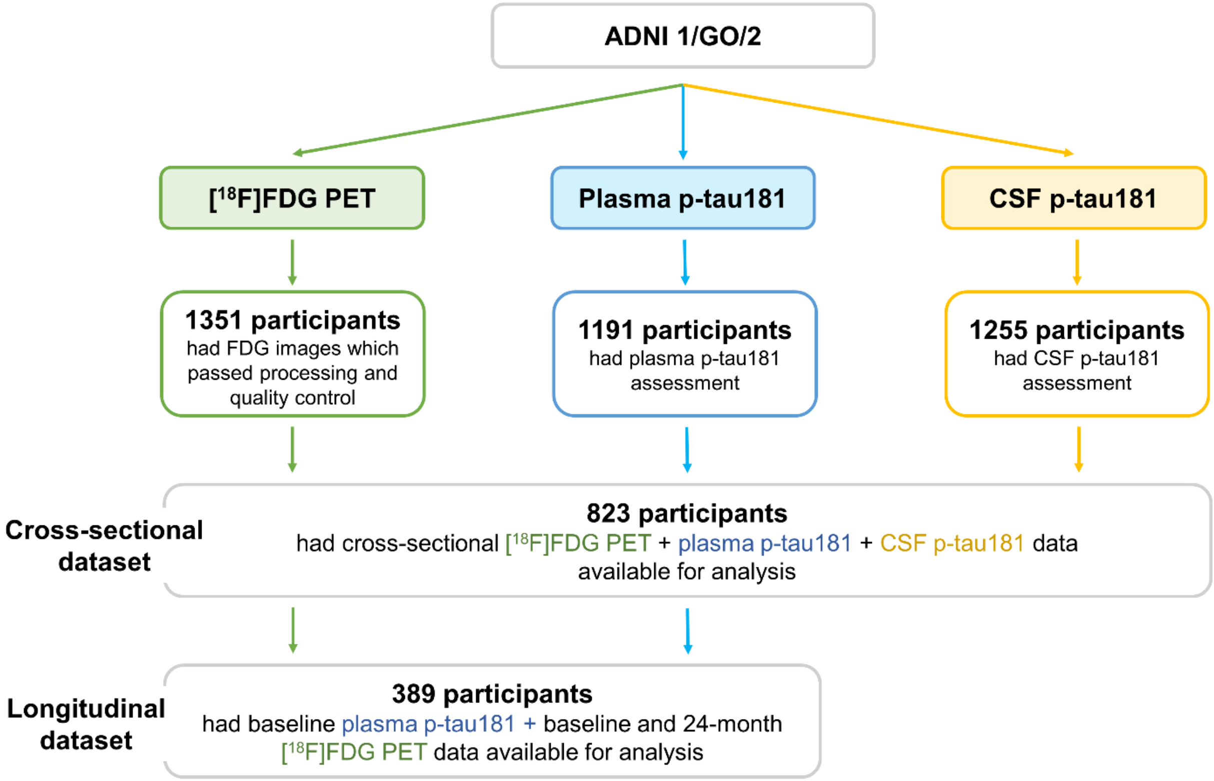

Supplementary Figure 1: This diagram visualizes how participants were selected from ADNI phases 1, GO, and 2 to be included in the cross-sectional and/or the longitudinal datasets. Inclusion of individuals in each dataset depended on the availability in ADNI and on the quality control of [^{18}F]FDG PET, plasma p-tau181 assessment, and CSF p-tau181 assessment.

Supplementary Figure 2: Voxelwise associations between CSF and plasma p-tau181 and [18F]FDG SUVR in all cognitively impaired individuals

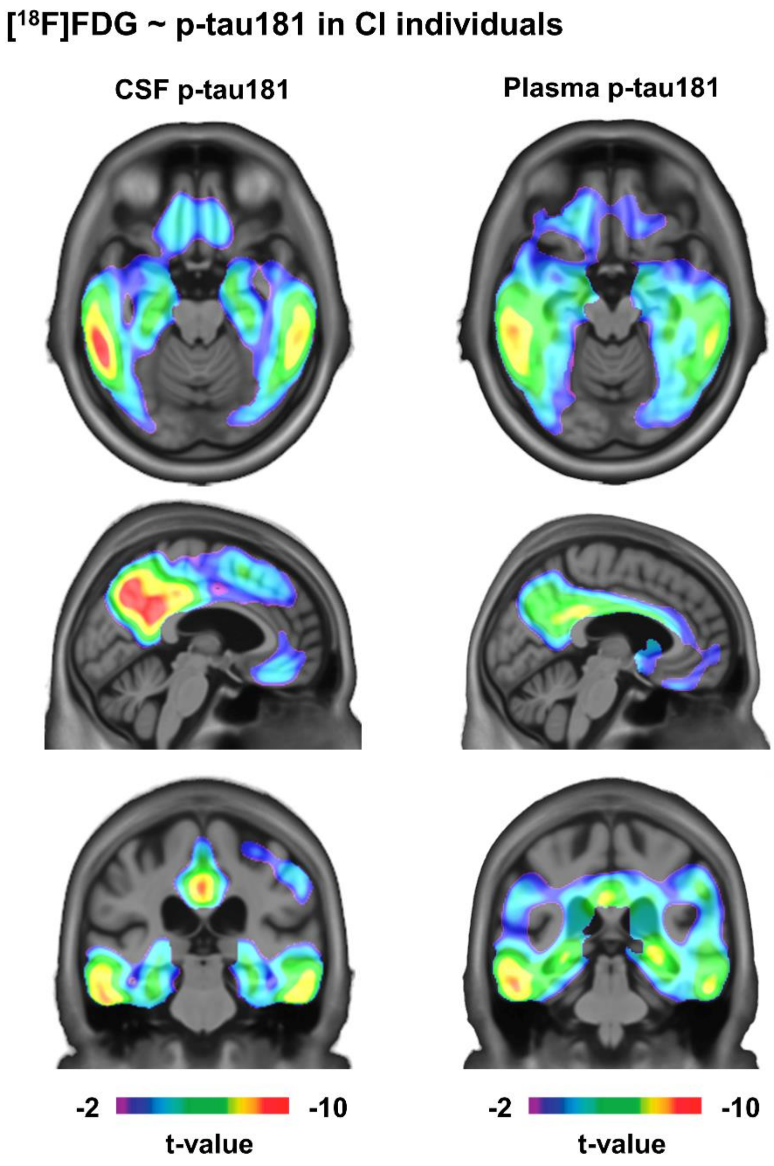

Supplementary Figure 2: Voxelwise linear regression models were used to assess associations between log-transformed CSF p-tau181 and plasma p-tau181 in all cognitively impaired (CI) individuals, adjusting for age and sex. Associations of CSF p-tau181 and plasma p-tau181 with [18F]FDG SUVR did not survive correction for multiple comparisons in CU individuals. In CI individuals, negative associations between CSF p-tau181 and [18F]FDG SUVR were observed bilaterally in the inferior temporal, posterior cingulate, precuneus, and orbitofrontal cortices (peak t-value -9.67). In the same group, negative associations between [18F]FDG uptake and plasma p-tau181 levels were found in the same brain regions as well as in the anterior cingulate (peak t-value -8.82).

Supplementary Figure 3: Average voxelwise annual rate of change in [^{18}F]FDG SUVR for participants in the longitudinal dataset

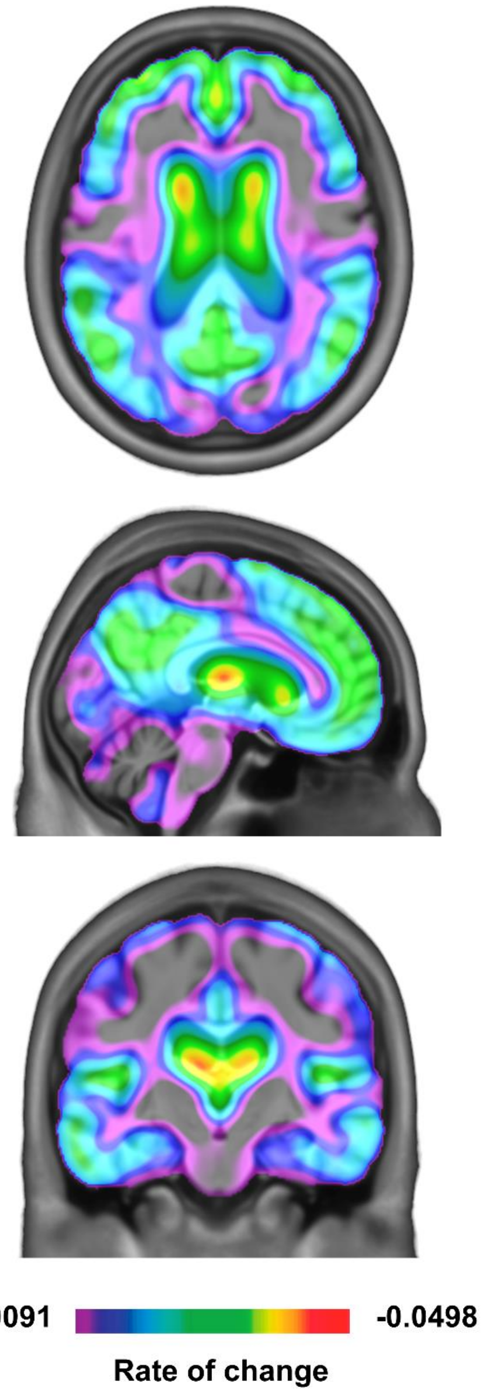

Supplementary Figure 3: Voxelwise annual rate of change in [^{18}F]FDG SUVR in participants in the longitudinal dataset (*n* = 389) ranged from 0.0091 to -0.0498, the latter of which represents a 2.8% decrease in [^{18}F]FDG SUVR compared to the mean value for all participants. Areas with the most prominent average decrease in [^{18}F]FDG SUVR were the posterior cingulate and precuneus, the medial and lateral temporal cortex, and medial frontal cortex.

Supplementary Figure 4: Correlation between plasma p-tau181 and change in [^{18}F]FDG SUVR according to cognitive and Aβ status

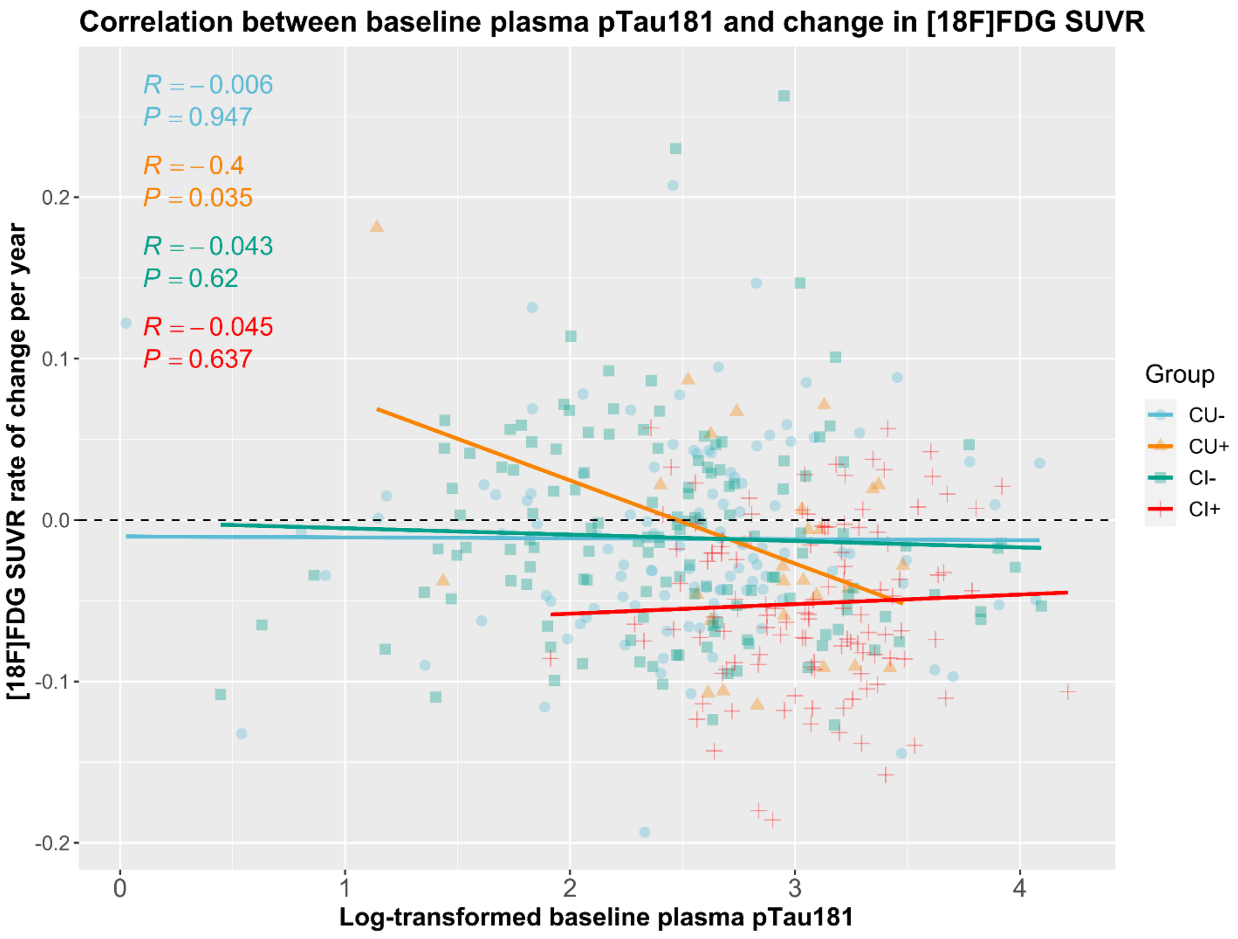

Supplementary Figure 4: We calculated Pearson's correlation coefficient (r) for associations between log-transformed baseline values of plasma p-tau181 (measured in pg/mL) and annual change in global [^{18}F]FDG SUVR for participants stratified by both their cognitive status (cognitively unimpaired (CU) or impaired (CI)) and Aβ status (+ or -). We found that correlations were only significant in the CU+ group (CU+, r=-0.4, *p*=0.035).
